# Comparison of two different recruitment maneuver patterns in ARDS patients

**DOI:** 10.1186/s40635-026-00854-z

**Published:** 2026-02-14

**Authors:** Davide Chiumello, Marialaura Montante, Pedro Wendel Garcia, Tapesh Bansal, Tommaso Pozzi, Silvia Coppola

**Affiliations:** 1 Department of Health Sciences, University of Milan, Milan, Italy; 2 Department of Anesthesia and Intensive Care, ASST Santi Paolo E Carlo, San Paolo University Hospital, Milan, Italy; 3 Coordinated Research Center on Respiratory Failure, University of Milan, Milan, Italy; 4 Institute of Intensive Care Medicine, University Hospital Zurich, Zurich, Switzerland; 5 Department of Critical Care Medicine, Paras Hospital, Gurgaon, India

**Keywords:** ARDS, Recruitment maneuver, Respiratory mechanics, Gas exchange, Electrical impedance tomography

## Abstract

**Background:**

The differential effects of the two most commonly investigated recruitment maneuvers (RMs), i.e., sigh recruitment and sustained inflation, have not been fully investigated yet. This study aimed to compare the effects of these two RMs on respiratory mechanics, gas exchange, and electrical impedance tomography (EIT)-derived lung volumes on mechanically ventilated ARDS patients.

**Results:**

This is a two-period two-sequence randomized crossover study. Two RMs were tested in randomized sequence: a sigh recruitment (one minute with a PEEP of 5 cmH_2_O, a driving pressure of 40 cmH_2_O at a respiratory rate of 10 bpm) and a sustained inflation maneuver (constant airway pressure of 40 cmH_2_O for 30 s). Following the application of the first RM, respiratory mechanics, hemodynamics and EIT-derived lung volumes were measured every 5 min for the following 30 min, while gas exchange was monitored every 15 min. After a 30-min washout period, intended to allow the lung to return to pre-recruitment steady-state conditions, the second RM was applied, and the same measurements were obtained. Twenty-three ARDS patients were enrolled; 13 patients underwent sigh recruitment as first RM and sustained inflation as the second RM, 10 patients underwent the opposite sequence. Patients who underwent both sigh recruitment or sustained inflation as first maneuver showed similar respiratory mechanics, hemodynamics, gas exchange and EIT-derived lung volumes before the RMs compared to patients who underwent sigh recruitment or sustained inflation as the second maneuver after the 30-min washout period. Independently from the order, after the application of each RMs, respiratory system, lung and chest wall mechanics, arterial oxygenation and EIT-derived lung volumes remained similar to baseline at all measurement timepoints from 5 to 30 min. Nine and 13% of patients increased PaO_2_/FiO_2_ over 20% from baseline 5 min after sigh recruitment and sustained inflation, respectively; a similar percentage was found after 30 min.

**Conclusion:**

Neither a sigh recruitment nor a sustained inflation maneuvers had clinically significant effect on respiratory mechanics, gas exchange, hemodynamics or EIT-derived lung volume distribution within the first 30 min after their application.

**Supplementary Information:**

The online version contains supplementary material available at 10.1186/s40635-026-00854-z.

## Introduction

Acute respiratory distress syndrome (ARDS) is a heterogeneous disease, with different degrees of lung collapse or consolidation and alveolar shunting, depending on the etiology, time of onset, fluid balance, amount of lung edema and lung and chest wall elastances [1, 2]. As a consequence, invasive mechanical ventilation is typically used to ensure adequate gas exchange and minimize ventilator-induced lung injury [3–5]. Nowadays, it is widely recommended that low tidal volumes and driving pressures be applied to limit lung stress and strain [6]. However, patients with ARDS can develop progressive alveolar collapse and hypoxemia related to the degree of hypoventilation [7, 8]. Quantitative lung CT scan analysis has shown that the amount of potentially recruitable lung (i.e., the amount of collapsed lung that can be re-expanded) can range from 5 to 60% of total lung weight [1, 7]. In order to reopen collapsed and atelectatic lung regions (*i.e.,* to recruit these areas) it is necessary to increase airway pressure during inspiration in order to overcome the opening pressure [7, 9].

Recruitment maneuvers (RMs) are used to provide sufficient airway pressure for a sufficient period of time to reopen collapsed and atelectatic lung regions [10–12]. RMs can be used to improve gas exchange and respiratory mechanics (*i.e.,* to ameliorate lung elastance) as part of a ventilator strategy or at the physician’s discretion in case of lung derecruitment [10]. The main side effect of RMs is hemodynamic impairment, characterized by a decrease in cardiac output of up to 60%, which is usually reversible within a few minutes of resuming mechanical ventilation [10, 12–14]. The latest European guidelines on ARDS management recommended against the use of prolonged high-pressure RMs to reduce mortality in ARDS patients (moderate level of evidence against) [6]. Although the routine application of RMs does not affect the outcome, their use reduces the need for rescue therapy and they should be used on a case-by-case basis for hypoxemic patients as a life-saving procedure [6, 15]. The most commonly used RMs are the extended sigh, in which an intermittent increase in airway pressure is applied, or the sustained inflation, which is achieved by applying a constant airway pressure by combing different amount of PEEP and tidal volume for a continuous amount of time [10, 14, 16–18]. The response to RMs may differ according to the type of RM, the underlying etiology and the extent and distribution of the disease within the lung [19, 20]. In addition, the airway pressure applied to the respiratory system during the RM, as well as its duration (*i.e.,* the inflation pressure–time product) can affect lung recruitment [21].

This study aimed to investigate the effects of two different RMs (an extended sigh and a sustained inflation at similar airway pressures and durations) on respiratory mechanics, gas exchange, hemodynamics and electrical impedance tomography (EIT)-derived lung volumes in patients with ARDS.

## Materials and methods

### Study population

This two-period two-sequence randomized crossover study was performed from December 2022 to December 2024 at the ICU of the ASST Santi Paolo Carlo, San Paolo University Hospital, Milan, Italy. All patients with ARDS as defined by the Berlin definition criteria were considered eligible for the study. Exclusion criteria were: mechanical ventilation for more than 3 days and hemodynamic instability, defined as ongoing hyperlactatemia or the need for a norepinephrine infusion of more than 0.2mcg/kg/min.

The study was approved by the institutional review board of the ASST Santi Paolo e Carlo (protocol number 218/206) and informed consent was obtained in accordance with the Italian regulations.

### Study protocol

Figure S1 summarizes the study protocol flow chart. Participants were randomized into treatment sequences (e.g., A-B or B-A) using a computer-generated permuted block algorithm with variable block sizes (2–4). This ensured balanced allocation and reduced predictability. The randomization list was prepared independently, and allocation concealment was implemented via a secure centralized system. Patients were ventilated according to a lung protective strategy with a tidal volume of 8 mL/kg of predicted body weight and a respiratory rate to maintain an arterial carbon dioxide between 40 and 60 mmHg. PEEP was set according to a decremental PEEP trial, as the best compromise between partitioned respiratory mechanics and gas exchange; this was chosen by the attending physician, who was not involved in the study. The inspired oxygen fraction (FiO_2_) was adjusted to maintain arterial saturation between 90 and 95%. All enrolled patients were deeply sedated with propofol and remifentanil and paralyzed with rocuronium to assure muscle relaxation. Before the randomization, an initial sigh recruitment maneuver (see below) was performed to remove the possible influence of possible confounders arising from routine clinical practice (e.g., daily nursing, airway suctioning, eventual disconnection, etc*.*), to homogenize lung aeration and mechanics and to achieve a uniform starting point for subsequent measurements of gas exchange and respiratory mechanics, before assessing the effects of the study interventions, as already performed in other clinical trials [1, 22, 23]. One hour later, the first recruitment maneuver was performed according to the randomized sequence. Ventilatory settings were then resumed as previously described, after which the patients were monitored for 30 min, as we hypothesized to see the effects of the investigated RMs in this timeframe. After the end of the 30-min monitoring period following the application of the first RM of the randomized sequence, ventilatory settings were maintained unchanged for a further period of 30 min (*washout* period), to minimize the influence of the previous RM and to allow the lungs to return to their pre-recruitment steady-state conditions before applying the second RM in the randomized sequence [24, 25]. This was necessary to allow the comparison of RMs independently of their application order. After the application of the second RM, patients were monitored as after the first RM.

### Recruitment maneuvers

The two recruitment maneuvers investigated were:

- a sigh recruitment maneuver, consisting of the application of 1 min of pressure-controlled ventilation with a PEEP of 5 cmH_2_O and a driving pressure of 40 cmH_2_O to reach a peak pressure of 45 cmH_2_O with a respiratory rate of 10 bpm with an inspiratory-to-expiratory ratio of 1:1 [1];
- a sustained inflation maneuver, consisting in the application of a constant pressure of 40 cmH_2_O for 30 s [16].

### Data collection

Patients were equipped with an esophageal balloon (Nutrivent, Sidam, Modena, Italy), which was inserted to the lower third of the esophagus, as previously described [26], to obtain esophageal pressure measurements. After placement, an end-expiratory airway occlusion test was used to ensure the correct positioning the catheter [27]. A dedicated 16-electrode EIT belt was placed around the patient’s thorax at the level of the fifth–sixth intercostal space, to obtain dynamic lung volume distribution data (PulmoVista® 500, Dräger, Lübeck, Germany).

After each recruitment maneuver, the following variables were obtained every 5 min for a period of 30 min:

- *Partitioned respiratory mechanics.* Airway plateau pressure (Pplat) and esophageal plateau pressure (Pes,insp) after a five-second long inspiratory hold; total PEEP and end-expiratory esophageal pressure (Pes,exp) after a five-second expiratory hold;
- *Hemodynamics.* Systolic, diastolic and mean blood pressures, central venous pressure and heart rate;
- *Dynamic lung imaging data.* End-expiratory lung impedance were collected for the whole lung and for four dimensionally equal ventral-to-dorsal regions of interests (ROIs, from ROI1 the most ventral to ROI4 the most dorsal) in 2-min-long recordings by EIT at a frame rate of 50 Hz [28]. For each EIT-derived variable, the correct value was assumed as the average value from 5 consecutive breaths at the end of the tracing.

Moreover, every 15 min, gas exchange was assessed by an arterial blood gas analysis, to obtain arterial pH, carbon dioxide (PaCO_2_) and oxygen (PaO_2_) partial pressures, bicarbonate concentration and base excess.

### Derived variables

See Supplementary Material for more details. Driving pressure, respiratory system elastance, chest wall and lung elastances, lung stress and mechanical power were calculated as previously described [20, 29].

### Statistical analysis

Continuous data are reported as mean ± SD or median [IQR], as appropriate; categorical data are reported as number (%). Sample size was estimated for a two-period crossover design with a continuous endpoint (change in end-expiratory lung impedance, EELI). Based on previous literature [30], given the crossover design and assuming a high within-subject reproducibility of EIT measurements, to detect a minimum clinically relevant difference of 100 ∆Z in end-expiratory lung impedance between the interventions, a minimum of 23 patients was required to ensure the study a power of 0.80 with a significance level of 0.05. To assess differences between patients receiving sigh recruitment or sustained inflation as first or second RM, Student’s t test or Wilcoxon–Mann–Whitney U test were used, as appropriate. One-way analysis of variance (ANOVA) for repeated measures or Friedman test were performed to assess differences within timepoints after each of the RM applied. A post hoc analysis with Bonferroni correction was performed for multiple comparisons. Fisher exact test was used to compare the proportions of patients considered as responders in terms of increase in PaO_2_/FiO_2_ ratio, using 20% increase from baseline as threshold to define responsiveness, 15 and 30 min after sigh recruitment or sustained inflation. To explore the temporal pattern of the responses after RM application, the time course of each variable was visually inspected; based on the observed trends, the appropriate modeling approach was selected. Specifically, changes over time and between groups were analyzed using mixed-effects models, with time and group (and their interaction) included as fixed effects and subject as a random effect. When the visual inspection suggested an approximately linear evolution over time, a linear mixed-effects model was fitted; in cases where the response showed a nonlinear pattern, a nonlinear mixed-effects model was applied. Model assumptions were checked graphically, and model fit was compared using likelihood-based criteria (AIC) when relevant. A p value of < 0.05 was considered as statistically significant. Statistical analyses and figures were performed using R Studio (RStudio. Integrated Development for R. RStudio, PBC, Boston, USA).

## Results

Twenty-three ARDS patients were enrolled after 1 [1-2] days of mechanical ventilation. At enrollment a mean tidal volume of 470 ± 50 mL and a PEEP level of 10 [8-10] cmH_2_O were applied (Table 1). Patients presented a mean PaO_2_/FiO_2_ of 149 ± 48 with a ventilatory ratio of 1.4 ± 0.4. The resulting mechanical power was 17.5 ± 5.7 J/min. Hemodynamic data at enrollment are presented in Table S1.

**Table 1:** Baseline characteristics of the study population according to the first recruitment maneuver (RM) administered (sigh recruitment *vs* sustained inflation) in the randomized study protocol

	Sigh recruitment n = 13	Sustained inflation n = 10	*p*
Age, *years*	68 [49 – 77]	68 [57 – 73]	0.687
Male sex, *% (n)*	69 (9)	6 (60)	0.685
Weight, *kg*	83 ± 12	68 ± 14	**0.016**
Body mass index, *kg/m*^*2*^	28 ± 5	25 ± 4	0.070
Tidal volume, *mL*	490 ± 50	450 ± 50	0.063
Respiratory rate, *bpm*	16 ± 2	18 ± 3	**0.027**
PEEP, *cmH*_*2*_*O*	10 [10–12]	10 [8–10]	0.221
Plateau airway pressure, *cmH*_*2*_*O*	23 ± 4	21 ± 3	0.306
Driving pressure, *cmH*_*2*_*O*	12 ± 3	12 ± 3	0.863
Respiratory system elastance, *cmH*_*2*_*O/L*	25 ± 7	25 ± 6	0.891
Chest wall elastance, *cmH*_*2*_*O/L*	7 ± 2	8 ± 5	0.634
Lung elastance, *cmH*_*2*_*O/L*	18 ± 8	18 ± 4	0.849
Lung stress, *cmH*_*2*_*O*	16 ± 5	15 ± 4	0.602
Mechanical power, *J/min*	18.1 ± 6.3	16.7 ± 5.1	0.612
Arterial pH	7.39 ± 0.06	7.36 ± 0.05	0.233
PaCO_2_, *mmHg*	47 [8, 16, 40–50]	50 [46 – 58]	**0.222**
Ventilatory ratio	1.2 ± 0.3	1.7 ± 0.3	**0.001**
PaO_2_, *mmHg*	82 ± 20	83 ± 12	0.939
PaO_2_/FiO_2_,	152 ± 56	144 ± 39	0.672
[HCO_3_^−^], *mMol/L*	27.2 ± 3.5	29.9 ± 3.2	0.068
Base excess, *mMol/L*	2.3 ± 4.1	4.5 ± 3.2	0.155
Systolic arterial pressure, *mmHg*	123 ± 21	123 ± 22	0.224
Diastolic arterial pressure, *mmHg*	58 ± 9	57 ± 12	0.765
Mean arterial pressure, *mmHg*	80 ± 11	79 ± 13	0.498
Heart rate, *bpm*	79 ± 19	79 ± 19	0.945
Central venous pressure, *mmHg*	11 ± 3	11 ± 3	0.771

*PEEP* positive end-expiratory pressure, *PaCO*_*2*_ arterial carbon dioxide partial pressure, *PaO*_*2*_ arterial oxygen partial pressure, *[HCO*_*3*_^*−*^*]* bicarbonate concentration

### Before the beginning of the study protocol

Thirteen patients underwent sigh recruitment as first RM and sustained inflation as the second RM, while 10 patients underwent the opposite sequence. Before the first RM (T0), patients who underwent sigh recruitment and sustained inflation as first RM had a mean tidal volume of 490 ± 50 and 450 ± 50 mL with a PEEP of 10 [10–12] and 10 [8–10] cmH_2_O, respectively (Table S2). Regarding respiratory mechanics, driving pressure, partitioned respiratory mechanics (respiratory system, lung and chest wall elastance) and mechanical power were similar (Table S2). The PaO_2_/FiO_2_ was not different, while the PaCO_2_ was significantly higher in the sustained inflation group (54 ± 8 vs 45 ± 7 mmHg, *p* = 0.023) due a higher ventilatory ratio (Table S2).

### Adequacy of 30-min washout period

Patients who underwent sigh recruitment as first maneuver showed the same respiratory mechanics, hemodynamics, gas exchange and EIT-derived lung volumes at T0 than patients underwent sigh recruitment as second maneuver after the 30-min *washout* period. The same occurred to patients who received sustained inflation as first maneuver compared with patients who received sustained inflation as second maneuver.

Moreover, respiratory mechanics, hemodynamics, gas exchange and EIT-derived lung volumes showed the same time-course in patients who received sigh recruitment as first RM compared with patients who received sigh recruitment as second RM. The same occurred for sustained inflation (Table S3).

### Effects of RMs independently from the order

**Fig. 1:**
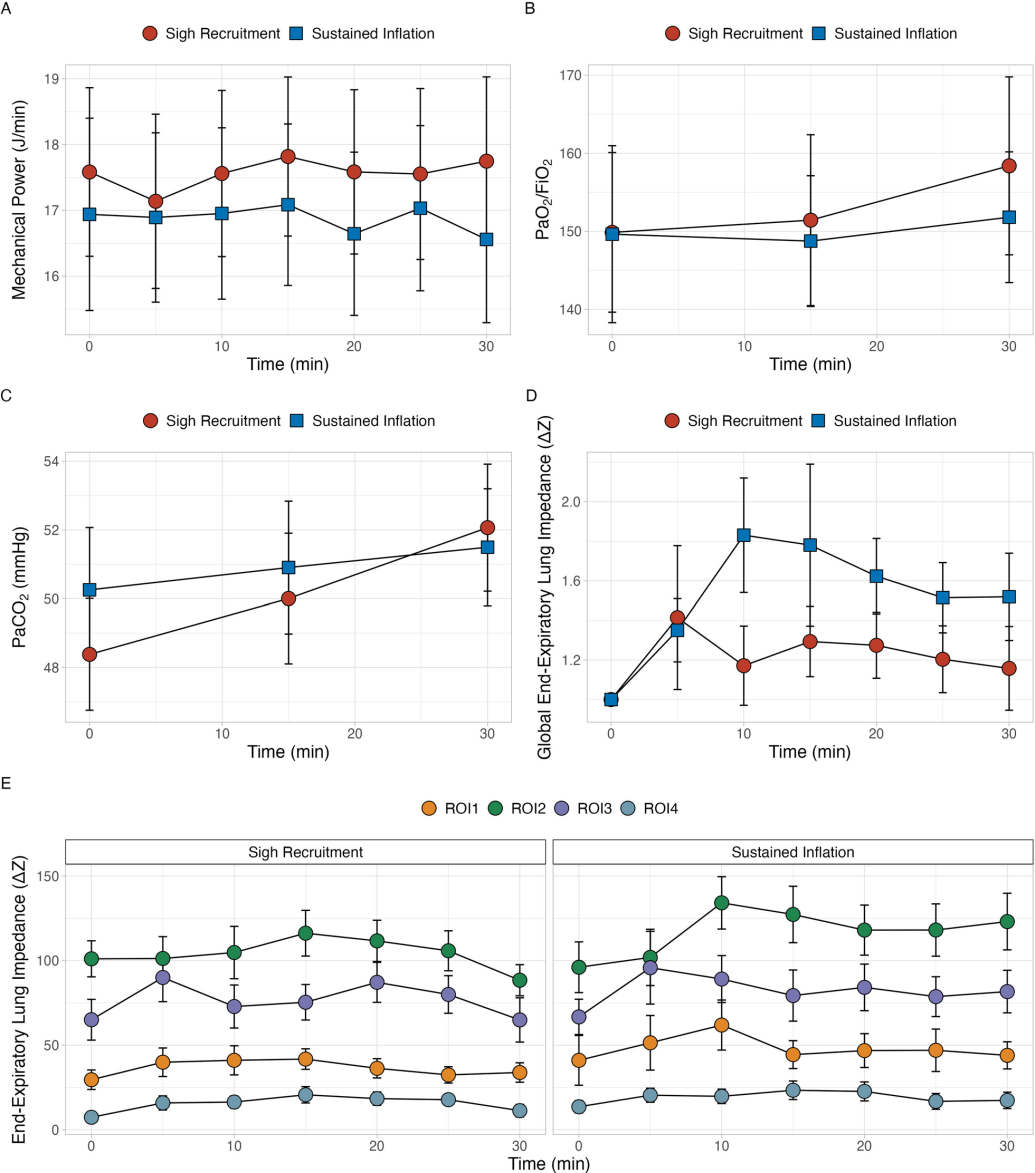
Time course of mechanical power (**A**), PaO_2_/FiO_2_ ratio (**B**), arterial carbon dioxide partial pressure (PaCO_2_—**C**), global end-expiratory lung impedance (EELI—**D**) and regional EELI (**E**) across regions of interest (ROIs) according to the recruitment maneuver (RM) applied within measurement timepoints (every 5 min to 30 min after the application of the RM for respiratory mechanics and EIT-derived lung volumes, and every 15 min after the application of the RM for gas exchange)

Driving pressure, as well respiratory system, lung and chest wall elastances were not modified by the application of RMs, remaining unchanged over measurement timepoints. Thus, also mechanical power was not affected by RMs (Table 2 and Fig. 1). Hemodynamics did not change after both RMs and remained stable throughout the study.

**Table 2:** Respiratory mechanics time-course according to recruitment maneuver (sigh recruitment or sustained inflation)

	Sigh recruiment n = 23	Sustained inflation n = 23	*p*_*RM*_	*p*_*TIME*_	*p*_*INT*_
Driving pressure, *cmH*_*2*_*O*					
T_0_	11 [10–14]	11 [9–12]	0.145	*0.083*	*0.259*
T_5_	11 [10–14]	11 [9–12]			
T_10_	11 [10–13]	11 [9–12]			
T_15_	11 [10–14]	11 [10–13]			
T_20_	11 [10–14]	12 [10–14]			
T_25_	11 [10–13]	11 [10–13]			
T_30_	11 [10–13]	11 [9–12]			
Respiratory system elastance, *cmH*_*2*_*O/L*					
T_0_	26 ± 7	23 ± 6	0.138	0.084	0.258
T_5_	26 ± 7	25 ± 7			
T_10_	26 ± 7	24 ± 6			
T_15_	25 ± 6	25 ± 7			
T_20_	26 ± 7	26 ± 7			
T_25_	25 ± 6	25 ± 7			
T_30_	26 ± 6	24 ± 6			
Chest wall elastance, *cmH*_*2*_*O/L*					
T_0_	5 [5–8]	6 [5–8]	0.758	0.063	0.307
T_5_	6 [3–8]	5 [2–7]			
T_10_	5 [3–8]	6 [4–7]			
T_15_	4 [3–7]	6 [5–7]			
T_20_	5 [3–7]	6 [5–7]			
T_25_	5 [3–7]	6 [5–8]			
T_30_	5 [3–7]	6 [5–7]			
Lung elastance, *cmH*_*2*_*O/L*					
T_0_	18 [1, 16–22]	17 [13–18]	0.098	0.939	0.936
T_5_	18 [16–22]	18 [16–22]			
T_10_	19 [1, 16–22]	17 [14–21]			
T_15_	18 [1, 15–22]	18 [15–22]			
T_20_	18 [1, 16–22]	19 [1, 17–24]			
T_25_	18 [1, 15–22]	18 [15–22]			
T_30_	19 [15–22]	18 [15–22]			
Lung stress, *cmH*_*2*_*O*					
T_0_	17 ± 4	15 ± 4	0.174	0.624	0.463
T_5_	17 ± 4	16 ± 5			
T_10_	17 ± 4	16 ± 4			
T_15_	17 ± 4	16 ± 4			
T_20_	17 ± 4	16 ± 4			
T_25_	17 ± 4	16 ± 4			
T_30_	17 ± 4	16 ± 4			
Mechanical power, *J/min*					
T_0_	17.6 ± 5.6	16.9 ± 5.7	0.958	0.093	0.076
T_5_	17.1 ± 5.8	16.9 ± 5.5			
T_10_	17.6 ± 5.5	17.0 ± 5.4			
T_15_	17.8 ± 5.3	17.1 ± 5.2			
T_20_	17.6 ± 5.3	16.6 ± 5.3			
T_25_	17.6 ± 5.7	17.0 ± 5.3			
T_30_	17.7 ± 5.6	16.6 ± 5.2			

*p*_RM_ between groups factor (sigh recruitment *vs* sustained inflation), *p*_TIME_ within group factor, *p*_INT_ interaction

Arterial oxygenation was not affected either by sustained inflation or sigh recruitment at each timepoints, while arterial carbon dioxide significantly increased after 15 and 30 from the application of both RMs without any differences between RMs (Table 3 and Fig. 1). Considering an increase in 20% in PaO_2_/FiO_2_ with respect to baseline as criterion to assess oxygen responsiveness to RMs, 9% and 13% of patients were considered responders after 15 min from the application of sigh recruitment and sustained inflation, respectively (*p* = 0.946); after 30 min, responders were 13% and 10% for sigh recruitment and sustained inflation maneuvers, respectively (*p* = 0.981).

**Table 3:** Respiratory mechanics time-course according to recruitment maneuver (sigh recruitment or sustained inflation)

	Sigh Recruiment n = 23	Sustained inflation n = 23	*p*_*RM*_	*p*_*TIME*_	*p*_*INT*_
PaCO_2_, *mmHg*					
T_0_	48 ± 8	50 ± 9	0.058	< 0.0001	0.106
T_15_	50 ± 9	51 ± 9			
T_30_	52 ± 9	52 ± 8			
Ventilatory ratio					
T_0_	1.4 ± 0.4	1.4 ± 0.4	0.634	< 0.001	0.118
T_15_	1.4 ± 0.4	1.4 ± 0.4			
T_30_	1.5 ± 0.4	1.5 ± 0.4			
PaO_2_/FiO_2_					
T_0_	150 ± 48	150 ± 53	0.972	0.134	0.574
T_15_	151 ± 51	149 ± 38			
T_30_	158 ± 54	152 ± 39			

*PaCO*_*2*_ arterial carbon dioxide partial pressure; PaO_2_: arterial oxygen partial pressure. *p*_RM_: between groups factor (sigh recruitment *vs* sustained inflation); *p*_TIME_: within group factor; *p*_INT_: interaction

The global end-expiratory lung impedance remained stable throughout the study (Table 4, Fig. 1). In addition, no redistribution of end-expiratory lung volume seems to be occurred from 5 to 30 min after each RM, as demonstrated by absence of interaction between the effect of time and each ROI (sigh recruitment: p = 0.941; sustained inflation: p = 0.392).

**Table 4:** Respiratory mechanics time-course according to recruitment maneuver (sigh recruitment or sustained inflation)

	Sigh Recruiment n = 23	Sustained inflation n = 23	*p*_*RM*_	*p*_*TIME*_	*p*_*INT*_
Global EELI, *ΔZ*					
T_0_	197 [139 – 281]	165 [108 – 297]	0.845	0.175	0.164
T_5_	240 [124 – 367]	183 [116 – 414]			
T_10_	221 [102 – 312]	274 [192 – 344]			
T_15_	238 [173 – 318]	211 [176 – 310]			
T_20_	240 [181 – 293]	204 [180 – 374]			
T_25_	237 [176 – 304]	218 [173 – 344]			
T_30_	203 [106 – 261]	251 [155 – 363]			
EELI ROI 1, *ΔZ*					
T_0_	28 [1, 8–39]	16 [1, 7–38]	0.636	0.724	0.924
T_5_	32 [1, 8, 13–48]	18 [13 – 57]			
T_10_	29 [1, 8, 13–52]	36 [23 – 80]			
T_15_	40 [8, 16, 23–52]	24 [16 – 74]			
T_20_	29 [1, 8, 16, 18–51]	30 [17 – 68]			
T_25_	32 [1, 8, 13–45]	29 [11 – 59]			
T_30_	27 [1, 8, 15–49]	35 [22 – 59]			
EELI ROI 2, *ΔZ*					
T_0_	100 [56 – 140]	77 [41 – 144]	0.902	0.679	0.158
T_5_	103 [41 – 146]	72 [46 – 146]			
T_10_	92 [58 – 148]	130 [89 – 161]			
T_15_	103 [68 – 144]	120 [67 – 155]			
T_20_	114 [67 – 133]	104 [76 – 178]			
T_25_	95 [70 – 148]	101 [66 – 165]			
T_30_	81 [67 – 113]	112 [84 – 166]			
EELI ROI 3, *ΔZ*					
T_0_	62 [31 – 107]	61 [28 – 93]	0.659	0.643	0.915
T_5_	71 [42 – 118]	74 [36 – 126]			
T_10_	79 [15 – 120]	83 [56 – 103]			
T_15_	59 [42 – 105]	76 [29 – 115]			
T_20_	79 [47 – 119]	89 [42 – 124]			
T_25_	89 [44 – 117]	82 [53 – 110]			
T_30_	58 [21 – 91]	90 [33 – 106]			
EELI ROI 4, *ΔZ*					
T_0_	5 [1–15]	11 [4–21]	0.179	0.400	0.759
T_5_	12 [4–21]	17 [1, 7–28]			
T_10_	9 [1, 5–27]	15 [1, 5–26]			
T_15_	15 [1, 5–27]	16 [1, 4–34]			
T_20_	11 [6–21]	19 [1, 7–33]			
T_25_	17 [1, 8–26]	14 [1, 4–23]			
T_30_	12 [4–19]	11 [1, 3–29]			

*EELI* end-expiratory lung impedance, *ROI* region of interest. *p*_*RM*_ between groups factor (sigh recruitment *vs* sustained inflation), *p*_TIME_ within group factor; *p*_INT_: interaction

## Discussion

The main finding of this study was that the application of two types of RMs (*i.e.,* a sigh recruitment or a sustained inflation) did not affect respiratory mechanics, gas exchange, hemodynamics and EIT-derived lung volumes either immediately after the maneuver or in terms of temporal patterns within 5 and 30 min afterwards in mild-moderate ARDS patients. However, considering a threshold for oxygen responsiveness as of 20% of increase in PaO_2_/FiO_2_, only a small proportion of patients were considered responders after 15 and 30 min from both RMs.

ARDS patients are characterized by the presence of collapsed lung regions, which can be caused by lung edema and the consequent superimposed pressure, by hypoventilation and by the administration of high oxygen fraction, resulting in reabsorption atelectasis [19]. Furthermore, the use of low tidal volumes in the context of a lung protective strategy can further worsen lung collapse and promote atelectasis [31, 32]. These factors result in a reduction in lung gas volume, contributing to the development of ventilator-induced lung injury [33]. Ideally, recruitment maneuvers (i.e., the transient increase in airway pressure) should not only increase the end-expiratory lung gas volume, but also recruit the lung by decreasing lung inhomogeneity. This should improve gas exchange by reducing shunt and avoid lung stress–strain [11, 34–37]. These theoretical advantages have led to the application of RMs since the 60’ to reverse the lung collapse, increase oxygenation and improve compliance during general anesthesia [38]. However, previous studies showed an heterogeneous response to RMs reaching an airway pressure between 45 and 60 cmH_2_O in terms of radiological recruitment and gas exchange [1, 7], depending on lung recruitability, which has been demonstrated to range from 5% up to 35–40% of the total lung weight, evaluated by CT scan [1]. Indeed, in poorly recruitable lungs, RMs might hyperinflate already ventilated regions, increasing the risk of barotrauma and promoting a decrease in cardiac output [39–41]. The RMs can induce hemodynamic impairment by increasing the intrathoracic pressure; thus, to minimize these effect, an airway pressure between 35 and 40 cmH_2_O for a period of 30–40 s have been suggested [42, 43].

A seminal study in ARDS patients showed that applying 3 sighs per minute for one hour, reaching an airway pressure of 45 cmH_2_O with high tidal volumes maintaining the same PEEP level significantly improved arterial oxygenation and respiratory mechanics [9] However, oxygenation and elastance, which significantly improved during the application of the sighs, returned to their baseline values within 20 min after sigh interruption and remained stable at 60 min [9].

According to the available data from three randomized controlled trials (RCTs) in which high pressure (*i.e.,* higher than 35 cmH_2_O) RMs were applied, no difference was found in terms of mortality and in barotrauma [8, 44, 45] as compared to ventilatory strategies without RMs. Indeed, the latest ESICM guidelines recommended against the use of high-pressure RMs to reduce mortality in patients with ARDS. In addition, it was also suggested against the routine use of brief high-pressure RMs to reduce mortality in ARDS patients (high level of evidence of no effect) [6]. The application of RMs should only be considered to reverse hypoxemia in the presence of derecruitment, for example after ventilator disconnection, suctioning, intubation or placement in the prone position [6]. The possible variables that could influence the response of RMs are the level of airway pressure, the application time, the ARDS etiology and the ventilatory settings (tidal volume and PEEP) after RMs [10, 21, 39, 46, 47]

The optimal level of airway pressure to be applied in ARDS patients depends also on the ratio between lung and chest wall elastances, which directly affects transpulmonary pressure, which is the pressure threshold for alveoli to be opened. For a similar airway pressure, the transpulmonary pressure can differ significantly [4]. Grasso et al*.* found that non-responders to RM presented a significantly higher chest wall elastance, resulting in a lower transpulmonary pressure. Additionally, non-responders exhibited a greater reduction in cardiac output due to a significant decrease in the pressure gradient for venous return [47].

Several studies have shown that RMs improve oxygenation and lung mechanics, particularly in patients in whom PEEP was increased after RM. These beneficial effects were not present when PEEP did not change as compared to the pre-recruitment ventilatory setting [12, 16, 46].

There are a variety of methods for performing RMs. The most common and straightforward approach is to set the ventilator in CPAP mode and increase PEEP to 30–40 cmH_2_O for 30–40 s [25, 35, 48]. Other methods proposed the increase in airway pressure by intermittently applying a high tidal volume (*i.e.,* sighs) or by simultaneously increasing PEEP and decreasing tidal volume to obtain a constant airway pressure [16–18].

A small randomized study found that, compared to sustained inflation at a similar airway pressure, a RM performed as sighs in pressure-controlled ventilation at 45 cmH_2_O significantly improved oxygenation with lower arterial carbon dioxide and hemodynamic impairment.

It has been suggested that the level of airway pressure applied by the ventilator, as well as the duration (*i.e.,* an adequate pressure–time product), affects lung recruitment. When a sustained inflation of up to 40 cmH_2_O was applied, the majority of the recruited lung volume was achieved within the first 10 s [49].

The present study evaluated two different RMs: sustained inflation and pressure-controlled ventilation, which both reached a similar peak airway pressure and inflation. Possible changes in lung volumes were assessed using electrical impedance tomography [50].

Thirty minutes after the application of both recruitment maneuvers, no differences in lung gas volume, arterial oxygenation or respiratory mechanics were found. The absence of any response could be explained by various factors, such as a low amount of lung recruitability (*i.e.,* a low amount of edema) or the presence of adequate PEEP levels, which already kept the lung “open”, the shorter duration of RMs and the evaluation timeframe (*i.e.,* during the RM/immediately after *vs* in the first hour after the RM). Furthermore, the application of RMs in ARDS patients with a focal rather than a diffuse morphology showed significantly lower lung recruitment and higher oxygenation [51]. A small increase in arterial carbon dioxide partial pressure was observed after the application of both sustained inflation and sigh recruitment; this minimal effect could be related to changes in lung perfusion induced by the increase in alveolar pressure during RMs.

Concerning the choice to apply a standardized recruitment maneuver before randomization, it could have been useful to homogenize lung aeration and mechanics before assessing the effects of different ventilatory strategies and to achieve a uniform starting point for subsequent measurements of gas exchange and respiratory mechanics, although it might also have decreased the potential positive effect of study interventions.

In conclusion, in patients with mild–moderate ARDS ventilated with moderate PEEP levels, the application of a RM, either as sigh recruitment or as sustained inflation, provided neither beneficial effects, in terms of improving oxygenation, respiratory mechanics and lung volumes, nor detrimental effects, in terms of hemodynamic impairment after 5 to 30 min.

## Supplementary Information

Supplementary material 1.

## Data Availability

The dataset used in this study is available upon reasonable request to the corresponding author.

## Abbreviations

ARDS: Acute respiratory distress syndrome
CT: Computed tomography
CPAP: Continuous positive airway pressure
PEEP: Positive end-expiratory pressure
ICU: Intensive care unit
EIT: Electrical impedance tomography
Pplat: Airway plateau pressure
Pes, insp: Esophageal plateau pressure
Pes, exp: End-expiratory esophageal pressure
PaCO2: Arterial carbon dioxide partial pressure
PaO2: Arterial oxygen partial pressure
FiO2: Inspired oxygen fraction
