# Supplementary material for: Comparison of two different recruitment maneuver patterns in ARDS patients
Source: Intensive Care Med Exp. 2026 Feb 14;14:18. doi: 10.1186/s40635-026-00854-z (PMC12905054; doi:10.1186/s40635-026-00854-z)
Supplement: Supplementary file 1 — Supplementary material 1. [file 40635_2026_854_MOESM1_ESM.docx]

**Comparison of Two Different Recruitment Maneuver Patterns in ARDS Patients**

***Supplementary Digital Material***

Davide Chiumello MD^1,2,3^, Marialaura Montante MD ^1,2^, Pedro Wendel Garcia MD^4^, Tapesh Bansal^5^, Tommaso Pozzi MD^1,2^ and Silvia Coppola MD^1,2^

*Supplementary Methods*

Driving pressure was calculated as [1]:

$$Driving Pressure=Plateau airway pressure-total PEEP$$

Plateau airway pressure and total PEEP were obtained with a five-second long inspiratory and expiratory holds, respectively, from the airway pressure tracing at the ventilator.

Respiratory system elastance was calculated as [1]:

$$Respiratory System Elastance=\frac{Driving Pressure}{V_{T}}$$

Where V_T_ is tidal volume (L).

𝛥Pes was calculated as:

$$\Delta Pes=Plateau esophageal pressure-End Expiratory Esophageal Pressure$$

Plateau esophageal pressure and end-expiratory esophageal pressure were obtained with a five-second long inspiratory and expiratory holds, respectively, from the esophageal pressure tracing.

Chest wall elastance was calculated as [1]:

$$Chest Wall Elastance=\frac{\Delta Pes}{V_{T}}$$

Lung elastance was calculated as [1]:

$$Lung Elastance=Respiratory System Elastance-Chest Wall Elastance$$

Lung stress was calculated as [2]:

$$Lung Stress=\frac{Lung Elastance}{Respiratory System Elastance}\times Plateau airway pressure$$

Mechanical power was calculated as [3]:

$$Mechanical Power=0.098\times V_{T}\times RR\times(Peak airway pressure-\frac{Driving Pressure}{2})$$

Ventilatory ratio was calculated as [4]:

$$Ventilatory Ratio=\frac{V_{E}\times PaCO_{2}}{0.1\times PBW\times37.5}$$

Where V_E_ is measured minute ventilation (L/min), PaCO_2_ is actual arterial carbon dioxide partial pressure and PBW is predicted body weight.

*Supplementary Tables and Figures*

**Figure S1**. Study protocol flow chart. PBW: predicted body weight; PaCO_2_: arterial carbon dioxide partial pressure; bpm: breaths per minute; RM: recruitment maneuver.

*
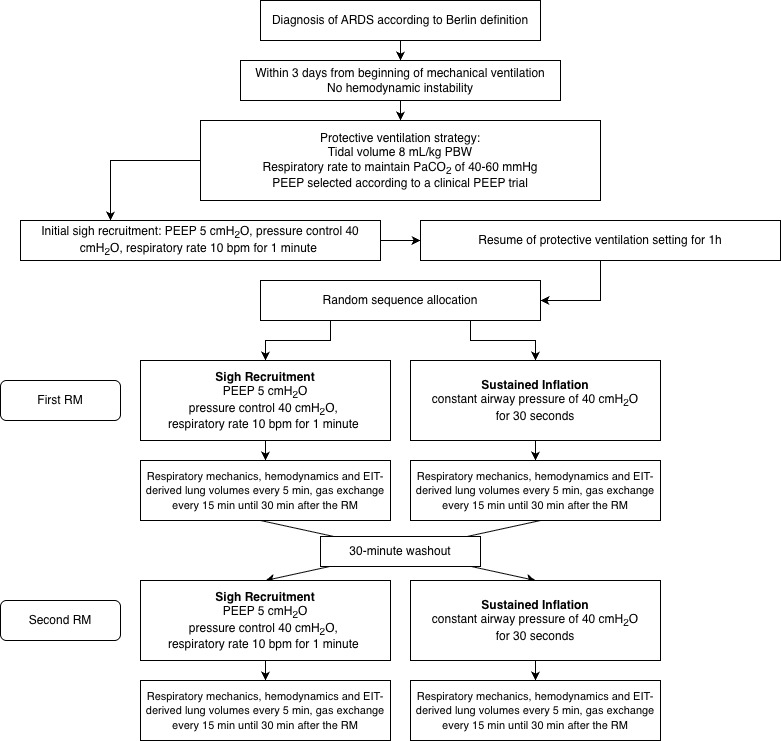
*

**Table 1**. Baseline characteristics of the whole study population. ARDS: acute respiratory distress syndrome; PEEP: positive end-expiratory pressure; PaCO_2_: arterial carbon dioxide partial pressure; PaO_2_: arterial oxygen partial pressure; [HCO_3_^-^]: bicarbonate concentration.

|  | n = 23 |
| --- | --- |
| Age, *years* | 68 [52 – 73] |
| Male sex, *% (n)* | 65 (15) |
| Weight, *kg* | 76 ± 15 |
| Body mass index, *kg/m^2^* | 27 ± 5 |
| Aetiology of ARDS, *% (n)*  Pneumonia  Sepsis  Other | 52 (12)  43 (10)  5 (1) |
| Origin of ARDS, *% (n)*  Pulmonary  Extrapulmonary | 52 (12)  48 (11) |
| Tidal volume, *mL* | 470 ± 50 |
| Respiratory rate, *bpm* | 17 ± 2 |
| PEEP, *cmH_2_O* | 10 [8 – 10] |
| Plateau pressure, *cmH_2_O* | 22 ± 3 |
| Driving pressure, *cmH_2_O* | 12 ± 3 |
| Respiratory system elastance, *cmH_2_O/L* | 25 ± 6 |
| Lung elastance, *cmH_2_O/L* | 18 ± 6 |
| Chest wall elastance, *cmH_2_O/L* | 7 ± 3 |
| Lung stress, *cmH_2_O* | 16 ± 4 |
| Mechanical power, *J/min* | 17.5 ± 5.7 |
| Arterial pH | 7.38 ± 0.06 |
| PaCO_2_, *mmHg* | 49 ± 8 |
| Ventilatory ratio | 1.4 ± 0.4 |
| PaO_2_, *mmHg* | 86 ± 21 |
| PaO_2_/FiO_2_ | 149 ± 48 |
| [HCO_3_^-^], *mMol/L* | 28.4 ± 3.6 |
| Base excess, *mMol/L* | 3.3 ± 3.8 |

**Table S2**. Baseline hemodynamic characteristics of the whole study population. PEEP: positive end-expiratory pressure; PaCO_2_: arterial carbon dioxide partial pressure; PaO_2_: arterial oxygen partial pressure; [HCO_3_^-^]: bicarbonate concentration.

|  | n = 23 |
| --- | --- |
| Systolic arterial pressure, *mmHg* | 122 ± 21 |
| Diastolic arterial pressure, *mmHg* | 58 ± 12 |
| Mean arterial pressure, *mmHg* | 80 ± 13 |
| Heart rate, *bpm* | 78 ± 19 |
| Central venous pressure, *mmHg* | 11 ± 3 |
| Amine requirement, *% (n)*  None  Single  *Norepinephrine*  Double  *Norepinephrine*  *Vasopressin* | 35 (8)  52 (12)  *100 (12)*  13 (3)  *100 (3)*  *100 (3)* |

**Table S3**. Respiratory mechanics, gas exchange, hemodynamics and EIT-derived measurements according to recruitment maneuver (RM) (sigh recruitment or sustained inflation) administration order (first *vs* second*)*. PEEP: positive end-expiratory pressure; PaCO_2_: arterial carbon dioxide partial pressure; PaO_2_: arterial oxygen partial pressure; EELI: end-expiratory lung impendance; ROI: region of interest.

|  | *Sigh recruitment* | | | *Sustained inflation* | | |
| --- | --- | --- | --- | --- | --- | --- |
|  | First  n = 13 | Second  n = 10 | *p* | First  n = 10 | Second  n = 13 | *p* |
| Plateau airway pressure, *cmH_2_O*  T_0_  T_5_  T_10_  T_15_ T_20_  T_25_  T_30_ | 23 ± 4  23 ± 4  22 ± 4  23 ± 4  23 ± 4  22 ± 4  22 ± 4 | 22 ± 3  22 ± 3  22 ± 3  22 ± 3  22 ± 3  22 ± 3  22 ± 3 | *0.453*  *0.750*  *0.795*  *0.628*  *0.718*  *0.674*  *0.628* | 21 ± 3  21 ± 3  21 ± 3  22 ± 3  21 ± 3  22 ± 3  21 ± 3 | 21 ± 3  21 ± 3  22 ± 3  22 ± 3  22 ± 3  22 ± 3  22 ± 3 | *0.912*  *0.958*  *0.787*  *0.991*  *0.672*  *0.939*  *0.778* |
| Driving pressure, *cmH_2_O*  T_0_  T_5_  T_10_  T_15_ T_20_  T_25_  T_30_ | 12 ± 3  11 ± 2  11 ± 2  11 ± 2  11 ± 2  11 ± 2  11 ± 2 | 12 ± 3  12 ± 3  12 ± 3  12 ± 3  12 ± 2  12 ± 3  12 ± 3 | *0.780*  *0.478*  *0.324*  *0.584*  *0.397*  *0.410*  *0.441* | 12 ± 3  12 ± 3  12 ± 3  12 ± 3  12 ± 2  12 ± 3  12 ± 3 | 10 ± 2  11 ± 2  11 ± 2  11 ± 3  11 ± 3  11 ± 3  12 ± 3 | *0.393*  *0.260*  *0.490*  *0.285*  *0.352*  *0.208*  *0.515* |
| Respiratory system elastance, *cmH_2_O*  T_0_  T_5_  T_10_  T_15_ T_20_  T_25_  T_30_ | 25 ± 7  24 ± 7  24 ± 6  24 ± 6  24 ± 6  23 ± 6  24 ± 6 | 25 ± 6  27 ± 7  26 ± 6  27 ± 6  28 ± 6  28 ± 7  26 ± 7 | *0.893*  *0.290*  *0.399*  *0.207*  *0.111*  *0.149*  *0.409* | 28 ± 7  28 ± 8  28 ± 7  27 ± 6  28 ± 7  27 ± 6  28 ± 7 | 21 ± 6  22 ± 7  22 ± 7  23 ± 7  24 ± 7  22 ± 6  23 ± 6 | *0.073*  *0.119*  *0.089*  *0.158*  *0.223*  *0.109*  *0.128* |
| Chest wall elastance, *cmH_2_O*  T_0_  T_5_  T_10_  T_15_ T_20_  T_25_  T_30_ | 7 ± 2  7 ± 3  6 ± 3  6 ± 4  5 ± 3  5 ± 2  5 ± 3 | 7 ± 5  5 ± 4  6 ± 4  6 ± 3  6 ± 3  6 ± 3  6 ± 3 | *0.586*  *0.437*  *0.678*  *0.646*  *0.428*  *0.415*  *0.649* | 5 ± 3  5 ± 3  5 ± 3  5 ± 3  5 ± 3  6 ± 3  5 ± 3 | 6 ± 2  6 ± 3  6 ± 3  6 ± 2  6 ± 3  6 ± 2  6 ± 3 | *0.616*  *0.804*  *0.888*  *0.267*  *0.607*  *0.672*  *0.495* |
| Lung elastance, *cmH_2_O/L*  T_0_  T_5_  T_10_  T_15_ T_20_  T_25_  T_30_ | 18 ± 8  18 ± 7  18 ± 7  18 ± 7  19 ± 6  18 ± 7  18 ± 7 | 18 ± 4  22 ± 8  20 ± 6  22 ± 7  22 ± 6  21 ± 6  20 ± 5 | *0.864*  *0.219*  *0.583*  *0.192*  *0.245*  *0.282*  *0.580* | 22 ± 7  23 ± 8  23 ± 8  22 ± 7  23 ± 8  22 ± 7  22 ± 8 | 15 ± 6  17 ± 8  17 ± 8  16 ± 7  18 ± 8  16 ± 6  16 ± 7 | *0.084*  *0.138*  *0.132*  *0.094*  *0.209*  *0.112*  *0.123* |
| Lung stress, *cmH_2_O*  T_0_  T_5_  T_10_  T_15_ T_20_  T_25_  T_30_ | 16 ± 5  16 ± 4  17 ± 5  16 ± 5  17 ± 4  17 ± 5  17 ± 5 | 15 ± 4  17 ± 4  16 ± 4  17 ± 4  17 ± 3  17 ± 3  16 ± 3 | *0.626*  *0.679*  *0.771*  *0.621*  *0.671*  *0.902*  *0.769* | 18 ± 3  18 ± 3  18 ± 4  18 ± 4  18 ± 4  17 ± 4  17 ± 4 | 15 ± 4  15 ± 6  16 ± 5  15 ± 4  16 ± 5  15 ± 4  15 ± 5 | *0.198*  *0.264*  *0.394*  *0.191*  *0.477*  *0.340*  *0.302* |
| Mechanical power *J/min*  T_0_  T_5_  T_10_  T_15_ T_20_  T_25_  T_30_ | 18.1 ± 6.3  17.8 ± 6.5  18.1 ± 6.3  18.1 ± 6.1  17.8 ± 6.3  17.9 ± 6.4  17.9 ± 6.4 | 16.7 ± 5.1  16.6 ± 4.6  16.7 ± 4.8  16.9 ± 4.3  16.4 ± 4.6  16.8 ± 4.6  16.2 ± 4.4 | *0.625*  *0.667*  *0.611*  *0.627*  *0.594*  *0.674*  *0.518* | 17.0 ± 5.0  16.5 ± 5.1  16.9 ± 4.8  17.5 ± 4.5  17.3 ± 4.6  17.2 ± 5.1  17.5 ± 4.9 | 17.2 ± 6.4  17.2 ± 6.5  17.2 ± 6.1  17.3 ± 6.2  16.9 ± 6.1  17.3 ± 6.3  16.9 ± 6.1 | *0.948*  *0.794*  *0.932*  *0.934*  *0.861*  *0.971*  *0.809* |
| pH  T_0_  T_15_ T_30_ | 7.39 ± 0.06  7.36 ± 0.05  7.37 ± 0.06 | 7.38 ± 0.06  7.36 ± 0.06  7.36 ± 0.06 | *0.250*  *0.629*  *0.603* | 7.38 ± 0.06  7.37 ± 0.06  7.37 ± 0.05 | 7.38 ± 0.06  7.37 ± 0.06  7.37 ± 0.05 | *0.885*  *0.774*  *0.930* |
| PaO_2_, *mmHg*  T_0_  T_15_ T_30_ | 82 ± 20  82 ± 19  88 ± 18 | 82 ± 12  80 ± 10  82 ± 10 | *0.943*  *0.842*  *0.377* | 81 ± 15  83 ± 17  84 ± 16 | 81 ± 15  83 ± 17  84 ± 16 | *0.990*  *0.462*  *0.881* |
| PaO_2_/FiO_2_  T_0_  T_15_ T_30_ | 153 ± 56  155 ± 62  166 ± 61 | 144 ± 39  142 ± 43  143 ± 36 | *0.682*  *0.578*  *0.301* | 147 ± 39  147 ± 38  149 ± 44 | 154 ± 64  154 ± 36  159 ± 42 | *0.740*  *0.653*  *0.587* |
| PaCO_2_, *mmHg*  T_0_  T_15_ T_30_ | 49 ± 8  50 ± 10  52 ± 9 | 50 ± 8  51 ± 8  52 ± 8 | *0.885*  *0.618*  *0.952* | 50 ± 9  51 ± 9  52 ± 8 | 50 ± 9  51 ± 9  52 ± 8 | *0.855*  *0.618*  *0.952* |
| Ventilatory ratio  T_0_  T_15_ T_30_ | 1.2 ± 0.3  1.2 ± 0.3  1.2 ± 0.3 | 1.7 ± 0.3  1.7 ± 0.4  1.7 ± 0.4 | *0.117*  *0.113*  *0.277* | 1.7 ± 0.3  1.7 ± 0.3  1.8 ± 0.3 | 1.2 ± 0.3  1.2 ± 0.3  1.2 ± 0.3 | *0.328*  *0.539*  *0.121* |
| Mean arterial pressure, *mmHg*  T_0_  T_5_  T_10_  T_15_ T_20_  T_25_  T_30_ | 80 ± 11  83 ± 13  83 ± 15  79 ± 13  81 ± 12  79 ± 11  79 ± 11 | 79 ± 12  78 ± 12  78 ± 13  79 ± 13  81 ± 16  80 ± 14  79 ± 14 | *0.717*  *0.278*  *0.445*  *0.913*  *0.918*  *0.920*  *0.994* | 79 ± 16  78 ± 14  79 ± 14  79 ± 15  78 ± 13  77 ± 12  78 ± 13 | 79 ± 11  80 ± 10  81 ± 11  81 ± 11  79 ± 11  80 ± 14  81 ± 11 | *0.991*  *0.664*  *0.610*  *0.728*  *0.843*  *0.621*  *0.509* |
| Global EELI, *ΔZ*  T_0_  T_5_  T_10_  T_15_ T_20_  T_25_  T_30_ | 244 ± 124  257 ± 150  231 ± 187  284 ± 153  260 ± 160  247 ± 125  181 ± 127 | 177 ± 86  233 ± 120  240 ± 107  215 ± 111  246 ± 86  221 ± 80  220 ± 85 | *0.182*  *0.684*  *0.893*  *0.245*  *0.802*  *0.568*  *0.415* | 179 ± 104  193 ± 107  241 ± 65  217 ± 60  272 ± 116  245 ± 91  279 ± 87 | 247 ± 214  326 ± 272  351 ± 235  314 ± 239  272 ± 196  273 ± 211  257 ± 188 | *0.369*  *0.163*  *0.170*  *0.227*  *0.997*  *0.700*  *0.750* |
| EELI ROI 1, *ΔZ*  T_0_  T_5_  T_10_  T_15_ T_20_  T_25_  T_30_ | 32 [9 – 45]  36 [15 – 51]  25 [13 – 56]  38 [26 – 65]  36 [15 – 64]  22 [13 – 48]  27 [15 – 58] | 21 [12 – 31]  18 [12 – 26]  32 [22 – 48]  27 [20 – 44]  37 [18 – 60]  24 [18 – 53]  40 [26 – 64] | *0.628*  *0.257*  *0.648*  *0.446*  *0.693*  *0.522*  *0.310* | 23 [8 - 39]  27 [6 - 43]  29 [16 – 50]  41 [27 – 49]  27 [23 – 44]  35 [21 – 41]  25 [15 – 39] | 13 [6 – 47]  18 [15 – 85]  44 [26 – 90]  19 [13 – 88]  28 [16 – 70]  31 [7 – 56]  34 [4 – 45]’ | *0.998*  *0.343*  *0.137*  *0.648*  *0.976*  *0.821*  *0.648* |
| EELI ROI 2, *ΔZ*  T_0_  T_5_  T_10_  T_15_ T_20_  T_25_  T_30_ | 114 ± 52  100 ± 68  96 ± 88  126 ± 73  115 ± 70  100 ± 63  76 ± 36 | 82 ± 58  86 ± 61  129 ± 39  111 ± 34  137 ± 59  122 ± 45  140 ± 48 | *0.186*  *0.610*  *0.266*  *0.558*  *0.439*  *0.360*  *0.223* | 84 ± 41  103 ± 57  118 ± 54  103 ± 54  107 ± 43  113 ± 50  104 ± 48 | 107 ± 82  115 ± 92  139 ± 94  140 ± 103  103 ± 78  114 ± 92  111 ± 94 | *0.446*  *0.740*  *0.545*  *0.323*  *0.904*  *0.961*  *0.819* |
| EELI ROI 3, *ΔZ*  T_0_  T_5_  T_10_  T_15_ T_20_  T_25_  T_30_ | 67 ± 36  96 ± 87  74 ± 70  86 ± 59  85 ± 66  94 ± 57  59 ± 17 | 61 ± 26  70 ± 36  69 ± 34  57 ± 40  78 ± 62  68 ± 50  82 ± 53 | *0.782*  *0.395*  *0.843*  *0.204*  *0.808*  *0.271*  *0.442* | 62 ± 38  83 ± 36  72 ± 51  62 ± 36  90 ± 45  62 ± 45  73 ± 36 | 71 ± 64  116 ± 113  106 ± 80  97 ± 88  89 ± 71  87 ± 61  81 ± 65 | *0.707*  *0.456*  *0.258*  *0.261*  *0.961*  *0.282*  *0.707* |
| EELI ROI 4, *ΔZ*  T_0_  T_5_  T_10_  T_15_ T_20_  T_25_  T_30_ | 16 ± 6  17 ± 14  18 ± 16  25 ± 15  23 ± 21  20 ± 19  20 ± 10 | 15 ± 12  13 ± 11  16 ± 15  21 ± 13  24 ± 21  25 ± 19  21 ± 12 | *0.423*  *0.982*  *0.802*  *0.262*  *0.640*  *0.894*  *0.823* | 4 [2 – 10]  12 [3 – 21]  15 [5 – 25]  10 [2 – 19]  11 [7 – 21]  12 [9 – 18]  12 [3 – 16] | 15 [5 – 24]  17 [12 – 34]  19 [8 – 34]  27 [6 – 56]  19 [7 – 35]  14 [8 – 20]  9 [3 – 42] | *0.292*  *0.313*  *0.497*  *0.186*  *0.483*  *0.821*  *0.648* |

**Table S4**. Hemodynamics time-course according to recruitment maneuver. PEEP: positive end-expiratory pressure; PaCO_2_: arterial carbon dioxide partial pressure; PaO_2_: arterial oxygen partial pressure; EELI: end-expiratory lung impedance; PLI: peak lung impedance; EILI: end-inspiratory lung impedance.

|  | Sigh Recruiment  n = 23 | Sustained inflation  n = 23 | *p_RM_* | *p_TIME_* | *p_INT_* |
| --- | --- | --- | --- | --- | --- |
| Systolic arterial pressure, *mmHg*  T_0_  T_5_  T_10_  T_15_ T_20_  T_25_  T_30_ | 123 ± 21  125 ± 20  127 ± 21  124 ± 21  127 ± 25  124 ± 21  124 ± 21 | 123 ± 22  123 ± 19  125 ± 21  124 ± 21  127 ± 25  123 ± 18  123 ± 20 | *0.860* | *0.399* | *0.673* |
| Diastolic arterial pressure, *mmHg*  T_0_  T_5_  T_10_  T_15_ T_20_  T_25_  T_30_ | 58 ± 9  59 ± 10  58 ± 11  57 ± 10  58 ± 10  57 ± 9  56 ± 10 | 57 ± 12  56 ± 9  58 ± 11  57 ± 10  57 ± 10  57 ± 10  58 ± 11 | *0.476* | *0.221* | *0.166* |
| Mean arterial pressure, *mmHg*  T_0_  T_5_  T_10_  T_15_ T_20_  T_25_  T_30_ | 80 ± 11  81 ± 13  81 ± 14  79 ± 13  81 ± 14  79 ± 12  79 ± 12 | 79 ±13  79 ± 11  80 ± 12  80 ± 13  79 ± 11  79 ± 12  80 ± 12 | *0.296* | *0.409* | *0.508* |
| Heart rate, *bpm*  T_0_  T_5_  T_10_  T_15_ T_20_  T_25_  T_30_ | 79 ± 19  78 ± 20  77 ± 20  78 ± 23  76 ± 20  73 ± 25  77 ± 20 | 79 ±19  77 ± 19  78 ± 18  77 ± 17  78 ± 17  79 ± 19  78 ± 18 | *0.174* | *0.072* | *0.130* |
| Central venous pressure, *mmHg*  T_0_  T_5_  T_10_  T_15_ T_20_  T_25_  T_30_ | 11 ± 3  11 ± 3  11 ± 3  11 ± 3  11 ± 3  11 ± 3  11 ± 3 | 11 ± 3  11 ± 3  11 ± 3  11 ± 3  11 ± 3  11 ± 3  11 ± 3 | *0.761* | *0.127* | *0.387* |

1. Henderson WR, Chen L, Amato MBP, Brochard LJ (2017) Fifty Years Research in ARDS.Respiratory Mechanics in Acute Respiratory Distress Syndrome. Am J Respir Crit Care Med 196:822–833. https://doi.org/10.1164/rccm.201612-2495CI

2. Gattinoni L, Carlesso E, Caironi P (2012) Stress and strain within the lung. Curr Opin Crit Care 18:42–47. https://doi.org/10.1097/MCC.0b013e32834f17d9

3. Gattinoni L, Tonetti T, Cressoni M, et al (2016) Ventilator-related causes of lung injury: the mechanical power. Intensive Care Med 42:1567–1575. https://doi.org/10.1007/s00134-016-4505-2

4. Sinha P, Calfee CS, Beitler JR, et al (2019) Physiologic Analysis and Clinical Performance of the Ventilatory Ratio in Acute Respiratory Distress Syndrome. Am J Respir Crit Care Med 199:333–341. https://doi.org/10.1164/rccm.201804-0692OC
